# P-171. Knowledge and acceptability of Fecal Microbiota Transplantation among patients and healthcare providers in Ethiopia

**DOI:** 10.1093/ofid/ofaf695.395

**Published:** 2026-01-11

**Authors:** Brandie Banner Shackelford, Michael H Woodworth, Kiya Kedir Kedir, Bizunesh Sintayehu, Ahmed Babiker, Workeabeba Abebe, Eyob Beyene Deyaso, Alemseged Abdissa, Monique Hennink

**Affiliations:** Emory University, Atlanta, GA; Emory University, Atlanta, GA; Armauer Hansen Research Institute, Addis Ababa, Adis Abeba, Ethiopia; Armauer Hansen Research Institute, Addis Ababa, Adis Abeba, Ethiopia; Rush University Medical Center, Chicago, IL; Addis Ababa University, Addis Ababa, Adis Abeba, Ethiopia; Addis Ababa University, Addis Ababa, Adis Abeba, Ethiopia; Armauer Hansen Research Institute, Addis Ababa, Adis Abeba, Ethiopia; Emory University, Atlanta, GA

## Abstract

**Background:**

Malnutrition and antimicrobial-resistant (AMR) infections are major causes of illness and death in low- and middle-income countries (LMICs), closely linked to the gut microbiome. Fecal microbiota transplantation (FMT) shows promise for treating AMR colonization and malnutrition, but little is known about its acceptability in LMICs. This study uses qualitative research to explore acceptability of FMT amongst healthcare providers and patients on FMT.

**Methods:**

Conducted in two Addis Ababa hospitals, the study purposively sampled patients (or caregivers of children) with bacterial infections and child malnutrition, along with healthcare providers. Focus group discussions (FGDs) were held with 53 participants: three groups with male patients, two with female patients, and three with mixed-gender healthcare providers. Amharic and English discussion guides covered FMT knowledge and acceptability, formulation preferences, and perceived barriers or facilitators. Data were translated, transcribed, and deidentified for thematic analysis using MAXQDA. Analysis involved the following iterative tasks: reading transcripts to identify emerging issues, developing a codebook, coding data, assessing inter-coder agreement, comparisons, categorization, and checks to ensure findings were grounded in the data.

**Results:**

Preliminary results highlighted limited knowledge of FMT among patients, while providers had varying baseline knowledge. Participants had varying views on whether more information would increase acceptability. Patients were primarily influenced by the opinion of their physician regarding its efficacy as well as cultural and religious factors. Providers reported that established medical guidelines and perceived patient adherence would influence whether they prescribe FMT. Participants said they’d consider adopting FMT if it was accessible in the local marketplace, affordable to patients, and other treatments failed.

**Conclusion:**

These emerging issues suggest that barriers to adopting FMT and other microbiota therapeutics in Ethiopia may be addressable in product formulation, design, and/or marketing.

**Disclosures:**

Ahmed Babiker, MBBS, MSc, Beckman Coulter Inc.: Advisor/Consultant

